# The oral microbiome in aging: a window into health and longevity

**DOI:** 10.1080/20002297.2025.2589648

**Published:** 2025-12-01

**Authors:** Zijun Yue, Chunhao Li, Fangxu Yan, Shuwen Guan, Yue Fan, Xingming Chen

**Affiliations:** a Department of Otolaryngology-Head and Neck Surgery, Peking Union Medical College Hospital, and Peking Union Medical College, Chinese Academy of Medical Sciences, Beijing, China

**Keywords:** Microbiology, oral microbiome, aging, gerontology, frailty, immunosenescence, inflammation

## Abstract

**Background:**

Aging is characterized by progressive physiological decline and increased susceptibility to age-related diseases. The oral microbiome, a complex community of microorganisms, has been increasingly recognized as a potential key player in the aging process.

**Objective:**

This review aims to explore and summarize the relationship between the oral microbiome and aging, with a specific focus on contrasting microbial changes in healthy and unhealthy aging populations.

**Design:**

We conducted a comprehensive review of the current literature to synthesize evidence on oral microbiome shifts during aging, the influencing factors, associations with age-related conditions, and potential interventions.

**Results:**

Evidence indicates that the composition of the oral microbiome changes with age, although findings on diversity are inconsistent, with reports of both increases and decreases in older adults. These shifts are influenced by factors such as diet, oral hygiene, and immune function. Unhealthy aging, including conditions like frailty, neurodegenerative diseases, and sarcopenia, is associated with distinct oral dysbiosis. Potential mechanisms linking the oral microbiome to aging include chronic inflammation and immunosenescence. Interventions targeting the oral microbiome, such as probiotics and dietary modifications, show promise in promoting healthspan.

**Conclusions:**

The oral microbiome is significantly altered during aging and is implicated in age-related health status. It represents a promising target for strategies aimed at promoting healthy aging. Future research should prioritize elucidating the functional mechanisms of oral microbiota and developing targeted microbiome-based interventions.

## KEY MESSAGES

Oral microbiome changes with age and are linked to frailty and diseases.

Chronic inflammation and immunosenescence are key underlying mechanisms.

Modulating the oral microbiome is a promising strategy for promoting healthy aging.

## Introduction

The life cycle's unavoidable physiological process of aging is marked by a loss in the body's resistance to stress, an increase in the risk of age-related disorders, and the deterioration of the physiological functions and structures of numerous organs and systems [1,2]. With the progression of global aging, the burden of this disease is expected to further intensify worldwide. Unhealthy aging is a significant factor contributing to a reduction in the human lifespan. Because aging results in a reduction in quality of life and a financial burden, many studies have been conducted to promote healthy aging and delay aging.

Numerous studies have explored the changes in the human microbiome during the aging process and identified it as a potential intervention target for delaying aging and promoting healthy aging [3–6]. Among the 14 hallmarks of aging proposed by López-Otín et al. [2], gut microbiome dysbiosis was first described in 2023 as one of the integrative hallmarks that explain aging phenotypes. The authors suggested that interventions aimed at restoring a 'younger' microbiome might extend a healthy lifespan. Moreover, thorough investigations of the oral‒gut axis have revealed that the microbiomes at the two extremities of the digestive system are not completely separate. In fact, the continuous colonization of the oral gut microbiota, as well as the connections between metabolites and immune responses, has been confirmed to play a role in the development of various systemic and age-related diseases [7]. Although research on the oral microbiome is still in its early stage compared to that on the gut microbiome, existing studies still indicate a link between the oral microbiome and aging.

This article is presented as a narrative review. A narrative approach was chosen to provide a comprehensive and integrative overview of the current evidence regarding the oral microbiome and aging, allowing for a focused discussion of potential mechanisms and intervention strategies.

The purpose of this review is to explore whether the oral microbiome, which serves as a common gateway for the microbiota of the respiratory and digestive systems, should be considered a target for predicting and delaying aging. We focus primarily on the changes in the oral microbiome during healthy aging, the characteristics of the oral microbiome in unhealthy aging states such as frailty and age-related diseases and the possible mechanisms underlying the association between the oral microbiome and aging. Finally, we summarize the current research findings and provide possible directions for microbiome-based aging interventions.

## Main text

The oral microbiome is composed of a variety of bacteria, viruses, fungi, and various protozoa, making it the second-largest microbial reservoir in the human body [8]. The establishment of the human oral microbiome begins at birth and undergoes the most dramatic changes during the first 3–5 years of life [9]. These changes are primarily associated with factors such as mode of delivery, breastfeeding [10], introduction of solid foods, and eruption of primary teeth [11]. During puberty, the oral microbiome experiences fluctuations influenced by sex hormones [12]. In adulthood, these changes tend to stabilize, with the 'core microbiome' in the oral cavity maintaining resilience over time within an individual [13]. Factors such as age, race, geographical location, dietary habits [14,15], physical activity, and oral conditions [16] (e.g. number of teeth and presence of oral diseases) can influence the composition and abundance of the oral microbiome. Numerous studies have shown that age itself is independently related to the variety and composition of the oral microbiome, even after adjusting for other factors. The baseline data and patterns of how the oral microbiome changes with age need to be more thoroughly elucidated and summarized to print a map for future research related to the oral microbiome. We focused mainly on the diversity of the oral microbiome, changes in the abundance of related taxa, and possible mechanisms underlying both healthy and unhealthy aging processes.

## Characteristics of the oral microbiome and related influencing factors in healthy aging

### Inconsistent findings exist on the diversity changes of the oral microbiome in healthy aging populations

The conclusions of existing studies on the diversity of the oral microbiome are summarized in Table 1.

**Table 1.** A summary of the patterns of age-related changes in the diversity and abundance of the oral microbiome across different studies.

Study subjects	Microbiome sample	Microbiome evaluation	Diversity changes	Abundance changes	References
General healthy population, age groups: <38, 38−51, 51−64, 64−77, and 77−80	Saliva	16S V4	*α*-diversity increases with age. *β*-diversity increases with age.	Positively correlated with age: *Comamonas, Phocaeicola abscessus* and *Anaeroglobulus germinatus*; Negatively correlated with age: *Veillonella, Haemophilus, Veillonella atypica/disparand Granulicatella adiacens/para-adiecens*	Wells et al. [17]
Hospital healthy population, age groups: young (22−26), elderly (65−74), and older (75−95)	Oropharyngeal swab	16S V3-V4	*α*-diversity higher in the elderly. *β*-diversity: the elderly show significant differences to the young.	Positively correlated with age: *unidentified_Saccharibacteria*; Negatively correlated with age: *Streptococcus, Oribacterium, Rothia,* and *Actinobacillus*	Liu et al. [18]
Dental clinic population, age groups: young (18–45), middle-aged (46–64), and elderly (65–94)	Saliva	16S V1-V3	*α*-diversity decreases with age. *β*-diversity: differences between the young and middle-aged, but no differences between the middle-aged and elderly.	Negatively correlated with age: taxa *Porphyromonas endodontalis*, *Alloprevotella tannerae (phylum: Bacteroidetes)*, *Filifactors alocis (phylum: Firmicutes), Treponema* sp*. (phylum: Spirochaetes), Lautropia Mirabilis (phylum: Proteobacteria)* and *Pseudopropionibacterium* sp.*_HMT_194 (phylum: Actinobacteria)*	Schwartz et al. [20]
Hospital healthy population, age groups: A (11−15), B (18−20), C (28−32), D (38−45), and E (50−65)	Saliva, gingival crevicular fluid, tongue dorsum swab	16S V3-V4	*α*-diversity decreases with age. *β*-diversity increases with age.	Positively correlated with age: *Eubacterium sulci, Stomatobaculum longum, Alloprevotella rava, Porphyromonas gingivalis, Abiotrophia defectiva, Porphyromonas endodontalis, Parvimonas micra, Dialister pneumosintes, Dialister pneumosintes, Tannerella forsythia, Treponema medium, Slackia exigua*; Negatively correlated with age: *Delftia, Escherichia/Shigella, Herbaspirillum, Bradyrhizobium, Brevundimonas, Pelomonas, Serratia, Feacalibacterium prausnitzii*	Liu et al. [21]
Community population, age groups: young (19−33), elderly (68−88), and centenarian (99−107)	Saliva	16S V3-V4;ITS 1	Bacterial: centenarians have lower *α*-diversity than both young and older adults. Fungal: no differences both in *α*-diversity and *β*-diversity.	Negatively correlated with age: *Haemophilus* spp.	Wu et al. [24]
Community population, age groups: longevity (90−102), semi-longevity (70−89), and elderly (55−70)	Saliva	16S V1-V9	*α*-diversity decreases with age. *β*-diversity: no difference between the longevity and the elderly.	Positively correlated with age: *Streptococcus, Peptostreptococcus* and *Saccharopolyspora*; Negatively correlated with age: *Prevotella 6* and *Pseudopropioni bacterium*	Ji et al. [28]
Community population, age groups: 20−40, 40−60, and >60	Supragingival plaque, buccal mucosa	shotgun	*α*-diversity: U-shaped trend with age. *β*-diversity: different among three groups.	Positively correlated with age: *Streptococcus anginosus* and *Gemella sanguinis*; Negatively correlated with age: *Neisseria,Propionibacterium, Lactobacillus, Bifidobacterium, Parascardovia* and *Mogibacterium*	Kazarina et al. [23]
Community population, age groups: 13−20, 20−30, 30−40, 40−50, 50−60, and >60−85	Saliva	16S V3–V4	*α*-diversity: U-shaped trend with age. The elderly have higher species richness and genetic diversity.	Positively correlated with age: genera *Anaeroglobus, Eikenella, Fretibacterium, Comamonas, Olsenella, Phocaeicola*, phylum *Synergistetes*; Negatively correlated with age: genera *Alloprevotella, Streptobacillus, Haemophilus, Prevotella, Granulicatella, Bergeyella*, phyla *Bacteroidetes* and *Proteobacteria*	Willis et al. [22]
Elderly (>80) and healthy control (20−39)	Saliva	16S V3-V4	*α*-diversity reduced in the elderly, but without statistical significance.	Bacterial genera enriched in the elderly: *Neisseria*; Bacterial species reduced in the elderly: *Prevotella melaninogenica*	Yamamoto et al. [99]

Some studies revealed that the *α*-diversity of the oral microbiome in older adults is higher than that in younger adults. A twin cohort study from the UK [17] proposed that age is an independent influencing factor for both *the α*-diversity and *β*-diversity of the oral microbiome, with the diversity of the saliva microbiome increasing with age. Further analysis indicated that the association between the oral microbiome and aging is driven by six taxa (Table 1). A study conducted by Liu et al. in Northeast China reached similar conclusions [18], reporting that the diversity of the oropharyngeal microbiome in elderly individuals is significantly greater than that in younger individuals, characterized by greater richness and evenness.

In contrast, a recent systematic review synthesizing six studies reported that healthy older adults generally exhibit lower oral microbiome diversity than younger controls do, supporting the notion that aging is accompanied by a reduction in microbial diversity and ecological resilience [19]. Similarly, Schwartz et al. [20] reported that the salivary microbiome *α*-diversity tended to decline with age. In their subsequent analyses, the authors suggested that this reduction was associated primarily with edentulism and the lack of dentures, which may diminish the availability of oral ecological niches. They also reported a significant difference in *β*-diversity between the young group (mean age = 33.72) and the elderly group (mean age = 72.98) but no significant difference between the middle-aged group (mean age = 56.59) and the elderly group. Liu et al. [21] investigated the microbiota at different oral sites (gingival crevicular fluid (GCF), tongue dorsum (TB), and saliva (SAL)) in healthy individuals of different age groups and reported a decreasing trend of *α*-diversity in aging mouths, while *β*-diversity showed an opposite increasing trend at all three sites. That is, the differences in the microbial community structure at these three sites are greater in the elderly. The authors further applied the most differentially abundant microbial taxa as biomarkers to distinguish between groups of different age ranges, achieving high accuracy in some oral sites (GCFs). However, the elderly group in this study was relatively young, with ages ranging from 50–65 years [21].

Additionally, some studies support a nonlinear relationship between age and the *α*-diversity of the oral microbiome, such as a U-shaped trend [22,23]. In other words, middle-aged people had the lowest *α*-diversity indices, with notable variances, compared to younger and older adults. The microbial communities of younger and older people, however, exhibit greater diversity of various types. The older group is characterized by greater species richness and genetic diversity, while no significant differences were found in terms of species evenness [22].

The findings on differences in *β*-diversity of the oral microbiome are also inconsistent. According to certain studies, as people age, the diversity of the oral microbiome makeup within communities tends to increase [17,21], which may be attributed to uncontrolled confounding factors such as immune variability, the number of teeth, and medication use. Other studies have revealed changes in the organization of microbial communities only between young and elderly individuals [18,20],while other investigations have not found any discernible differences in the makeup of communities between the two groups [24].

Why do seemingly comparable studies report rising, falling, or static oral *α*-diversity with age? The answer may lie in a tangle of methodological and biological modifiers: ancestry, geography, diet, chronological cut-offs for 'elderly', baseline health, sample type (saliva, plaque, or swab), sequencing variable regions, and even the number of remaining teeth all shape the microbial profile detected. Because each investigation balances these factors differently, apparent contradictions across cohorts are almost inevitable. For instance, dietary practices in different regions can lead to slight variations in the composition of the 'core microbiome' [25]. The Mediterranean diet has been shown to reduce the abundance of periodontal pathogens in saliva [26]. Additionally, some studies classify individuals over 50 years old as the 'elderly group', which may mask changes truly associated with aging [21]. Moreover, the sources of the study populations differ: community-based cohorts and clinical samples may introduce selection bias in terms of health status. Finally, different variable regions of the 16S rRNA gene have varying amplification efficiencies for bacteria, which can also impact the accuracy of taxonomic identification (Table 1).

Another category of diversity studies that warrants separate discussion is centenarians research, a unique population in which people seek to uncover the secrets of longevity. Current studies on the microbiome of centenarians have focused mainly on the gut microbiome, indicating that the gut microbiome composition of extremely long-lived individuals is similar to that of younger individuals and differs from the microbial changes observed in the general elderly population [27]. In recent years, several studies have explored the characteristics of the oral microbiome in centenarians. The evidence currently available indicates that the *α*-diversity of the oral microbiome in centenarians is substantially lower than that in younger and older adults, although there are no statistically significant variations in *β*-diversity across the categories [24]. A study from Northeast China [28] compared the oral microbiome of centenarians with that of the general elderly population and reached similar conclusions. The age-related saliva microbiome in extremely long-lived individuals exhibited a decline in bacterial richness and evenness, with no significant differences in *β*-diversity between the two. Overall, research on the oral microbiome of centenarians is still limited and requires more cross-sectional and prospective cohort studies to explore the changes in the oral microbiome in this unique population.

Furthermore, the association between the oral microbiome and biological age—a tool for assessing the body’s internal functional and aging status—has also been elucidated. A study based on the National Health and Nutrition Examination Survey indicated that increased oral microbiome diversity may be associated with slower biological aging [29]. However, further evidence is needed to substantiate the robustness of this conclusion.

### The abundance of oral microbial taxa changes during aging

We compiled key microbial taxa that change with age from multiple studies (Table 1). The taxonomic levels at which data are provided vary, and the results differ significantly. However, changes in abundance with age do not necessarily imply that specific microbial taxa significantly influence aging. These changes in abundance may represent an advantage in the changing oral environment and ecological niches of the elderly, or they may offer ecological niches for opportunistic infections. Therefore, changes in abundance alone cannot be used to determine the role of a microbial group in aging. The following sections will further review representative microbial taxa that are currently considered to potentially participate in the aging process or affect the health status of the elderly.

Several studies have indicated that aging is often accompanied by shifts in the composition of the oral microbiome, characterized by a decreased relative abundance of core commensal taxa and an increased abundance of potentially pathogenic species associated with gingivitis and periodontal disease [22]. However, the direction and magnitude of these changes may vary across populations and study designs. In individuals with exceptional longevity, the oral microbiome is characterized by a decrease in the abundance of core microbial taxa (such as *Neisseria*) and an increase in the abundance of some secondary core taxa. Certain genera among these secondary core taxa have a notable abundance of long-lived individuals and could make up a distinctive microbial profile [23]. *Porphyromonas gingivalis*, a typical periodontal pathogen, is associated with multiple aspects of aging, including neurodegenerative diseases [30], systemic inflammation [31], and cellular senescence [32]. The age-predicting model constructed by Huang et al. is characterized by elevated levels of *Porphyromonas gingivalis*[33]. Conversely, a conclusion drawn from modeling assessments of 'oral microbiome age' indicated that an increase in bleeding sites associated with periodontitis could accelerate the aging of the oral microbiome [34], which provided a bidirectional association between increased abundance of periodontal pathogens and age.

Second, opportunistic pathogenic genera, such as *Mycoplasma*, *Selenomonas*, *Tannerella*, and *Alistipes*[35], are enriched in aged saliva samples from healthy elderly individuals. The genus *Tannerella* is among the commonly detected oral taxa in periodontitis patients [36], which aligns with the increasing prevalence of periodontitis with age. *Streptococcus anginosus* and *Gemella sanguinis* are two additional opportunistic oral infections that have been found to be enriched in elderly individuals in certain studies. These two bacteria are positively associated with age in samples of the buccal mucosa and supragingival plaque [23]. A study of the subgingival microbiome in postmenopausal older women [37] revealed that the abundance of several opportunistic pathogens, such as *Fusobacterium nucleatum*, *Veillonella dispar*, *Streptococcus oralis*, and *Veillonella parvula*, was linked with age [38]. Another well-known probiotic, *Lactobacillus*, was found to be more abundant in the oral cavity of healthy elderly individuals compared to younger people [39]. *Lactobacillus* has a dual role in the oral cavity: Under healthy conditions, moderate acid production can inhibit the overgrowth of certain pathogens (such as some strains of *Streptococcus mutans*) and compete for growth space. However, under unfavorable conditions (such as a consistent high-sugar diet), *Lactobacillus* can proliferate excessively, leading to enamel demineralization and increasing the risk of dental caries in the elderly [40]. In the study conducted by Ji et al. [28], the abundance of core oral bacterial genera in long-lived and elderly groups decreased with age, with only two exceptions, *Streptococcus* and *Prevotella*. Additionally, the authors examined the features of oral-gut microbial translocation in individuals with longer lifespans and discovered that bacterial exchange between the oral and gut environments did not strictly follow a linear correlation with age. This suggests that individuals with longer lifespans may be able to carry strains of bacteria that promote gut health through the oral-gut axis, though the precise mechanisms of this 'potential' are still unknown [28]. Willis et al.[22] proposed that the increased oral microbiome *α*-diversity in elderly individuals could be attributed to an increase in the variety of low-abundance taxa. These authors speculated that this phenomenon is related to the decline in oral immune capacity in elderly individuals—the so-called 'immunosenescence', which makes the older oral cavity more susceptible to colonization by low-abundance opportunistic pathogens.

Apart from the increase in opportunistic infections, alterations in the oral microbiome are also linked to some aging-related physiological changes. In the study by Schwartz et al. mentioned earlier [20], after controlling for edentulism and other confounding factors, aging itself was associated with a lower abundance of the taxa *Porphyromonas endodontalis*, *Alloprevotella tannerae*, *Filifactor alocis*, *Treponema*, *Lautropia mirabilis*, and *Pseudopropionibacterium* sp. *HMT_194*. Most of these taxa are known to reside on or near the tooth surface, suggesting that studies of the oral microbiome in elderly individuals may need to control for tooth count. In another study [41], although the authors did not observe significant differences in the levels and proportions of the oral microbiome with age, they still found that older adults with healthy gums tended to have higher levels of some subspecies of *Fusobacterium nucleatum*. Given that prior research has indicated that species of the genus *Fusobacterium* can offer some protection against dementia by colonizing subgingival niches and preventing the settlement of more inflammation-inducing pathogens, this may be connected to the higher percentage of gingival recession and root exposure in the elderly group in their study [42]. Research on human oral function [43] also indicates that the increased viscosity of saliva due to decreased salivary gland function in the elderly is detrimental to oral health, potentially reducing the clearance of oral pathogens and increasing the risk of infectious diseases in the elderly, which may promote oral microbial dysbiosis.

A significant question is raised: When studying the relationship between the oral microbiome and aging, is it necessary to account for these physiological changes associated with age? The multi-system functional deterioration that occurs with aging frequently includes several characteristics, which may not always signify the existence of a disease state. However, when comparing the divergent conclusions of different studies, documenting such differences seems necessary. Clarifying the explanatory effect of various aging-related changes on alterations in the oral microbiome will provide momentum for explaining these different conclusions.

Overall, a variety of factors affect the oral microbiota of healthy older people. Even after adjusting for as many confounding variables as feasible, distinct oral microbiome traits that are linked to aging on their own remain. Models that predict actual physiological age have partially validated these traits, which serve as a guide for determining whether the observed variations in the oral microbiome are caused by diseases or aging.

## The oral microbiome in unhealthy aging and frailty

In light of the earlier review, we propose that physiological aging' of the oral microbiome occurs, which means that the microbiome naturally changes with age regardless of whether an individual is 'healthy'. Moving forward, we attempt to explore the characteristics of the oral microbiome in individuals experiencing unhealthy aging and age-related diseases.

### Differences in the oral microbiome between healthy and unhealthy elderly populations

Initially, some research contrasted the oral microbiome traits of older adults in generally healthy and unhealthy groups. In a prospective case‒control study by Singh et al. [44], individuals with unhealthy aging (NHA) had lower *α*-diversity of oral microbial communities compared to those with healthy aging (HA). While *Haemophilus, Fusobacterium*, and *Capnocytophaga* were less common in the NHA group, *Neisseria* was comparatively less common in the NHA group among the most numerous genera. Their definition of 'unhealthy' was based on participants self-reporting the presence of at least one major illness (cancer, neurological diseases, cardiovascular diseases, chronic lung disease, diabetes/diabetic complications). *Neisseria* is an important commensal species in the oral cavity that normally competes with pathogens for resources and ecological niches to maintain oral homeostasis [45]. Moreover, *Haemophilus* has been found to gradually decrease with age [17]. Therefore, is the oral microbiome profile of the ill group just an extension of the pattern seen in the alterations in the oral microbiomes of older people who are typically healthy? That is, does the biological age of the oral microbiome of older people of the same age increase as a result of ill states? Biological age [46] is typically calculated based on chronological age and various biomarkers, reflecting the relationship between an individual's physiological state and chronological age. It has been used to assess mortality risk and health status. However, the oral microbiome has not yet been incorporated into biological age assessments, and further research is needed to explore the potential of the oral microbiome in biological age evaluation.

### Microbiome changes associated with frailty

Studying aging by concentrating on a specific disease appears incomplete because aging is a physiological process that involves several systems throughout the body. The systemic state of frailty is currently the focus of numerous studies on unhealthy aging. Frailty [47,48] is a common biological syndrome in the elderly characterized by reduced physiological reserves and resistance to stressors, accompanied by increased vulnerability to adverse health outcomes such as falls, disability, and hospitalization. The state of frailty does not necessarily imply the presence of disease or disability but increases the likelihood of their occurrence [49]. While disability can result from the breakdown of a single system, frailty involves many dysfunctional systems. In other words, frailty is intrinsically unstable, whereas disability is the opposite [50]. This uncertainty provides the possibility for primary and secondary prevention of diseases.

Numerous studies indicate that poor dental health may increase an older adult's risk of frailty. In Asian and European populations, reduced chewing ability is associated with higher incidence of frailty and mortality risk [51–53]. At the microbiome level, a study of hospitalized elderly individuals revealed that those with chewing difficulties presented an increased abundance of *Lactobacillus* in saliva and reduced overall microbial diversity, indicating that oral functional decline may indirectly trigger frailty by altering microbial ecology [54]. Further comprehensive analyses have demonstrated a substantial correlation between frailty and conditions such as decreased tooth count, oral dysfunction, and problems of salivary production [55–58]. These factors may also affect oral microbial homeostasis through impaired nutrient intake or changes in local ecological niches. In summary, the above studies emphasize that a decline in oral function [34] and microbial imbalance may jointly mediate the development of frailty, which deserves integrated assessment in future research.

The study of oral health conditions may lead us to the concept of 'oral frailty (OF)'. The number of remaining teeth, chewing capacity, oral transverse movement, tongue pressure, difficulty eating hard meals, and difficulty swallowing tea or soup were some of the parameters used by Kimura et al. [58] to determine whether OF was present [59]. They found that individuals meeting the criteria for OF had a higher proportion of *Prevotella* in their salivary microbiome. The contribution of this genus to pneumonia mortality in elderly individuals has been confirmed [60], and it has the potential to become a diagnostic biomarker for the early detection of OF, allowing early intervention and management to reduce the risk of its progression to systemic frailty [61]. In addition to bacterial research, some studies have examined the connection between oral fungal species and OF, revealing that OF is associated with salivary *Candida* carriage [62]. However, owing to various limitations of sequencing and species identification technologies, studies on fungi, viruses, and protozoa in the oral microbiome are still rare. It is difficult to form a mature perspective on the interactions between different categories of microorganisms in the oral microbiome and their impact on aging and frailty.

However, OF does not necessarily coexist with systemic frailty. Although some studies have suggested that the persistence of OF may increase the risk of systemic frailty in older adults, younger populations can also experience OF. The features of the oral microbiome in systemically weak elderly people as opposed to healthy elderly people are thus the subject of our next discussion.

#### Differences exist in the oral microbiome between systemically frail and healthy elderly individuals

In a comparative study between nursing home residents (*n* = 15) and community-dwelling older adults [63], the former (who had a significantly higher proportion of frailty) showed that *Haemophilus*, *Bacilli*, *Selenomonas*, *Veillonella*, *Actinomyces* and *Streptococcus* were more prevalent in their oral cavities than *Prevotella*, *Leptotrichia*, *Campylobacter* and *Fusobacterium*. Among the taxa with increased abundance, *Streptococcus* and *Haemophilus* have been associated with an increased risk of aspiration pneumonia in the elderly [64]. Two studies from the UK reached similar conclusions [17,65], suggesting that frailty is associated with a loss of richness and reduced diversity in the oral microbiome of older adults. In relation to microbiomes in other body sites, the loss of diversity in the gut core microbiome has also been reported to be associated with the occurrence of frailty [3]. The interpretation of the results may be impacted by the study's small sample size (*n* = 15) and failure to account for the confounding factor of participants' gingival condition [63]. Another study comparing inhabitants of nursing homes and those living in the community revealed notable differences in the oral microbiome associated with illness risk and antibiotic resistance (e.g. methicillin-resistant *Staphylococcus aureus*) [66]. It has been claimed that alterations are linked to frailty rather than chronological age when the contributions of age and frailty to these changes are analyzed independently.

Small sample numbers that restrict the interpretability of subgroup analyses, such as the relationship between sex and the oral microbiome, are a common problem among the aforementioned studies. In a large-sample study from Canada (*n* = 1357) [67], the authors found that the impact of age and frailty on the oral microbiome differed by sex. Salivary microbiome *β*-diversity was significantly associated with age across the entire cohort, while *α*-diversity metrics presented a more pronounced negative correlation with frailty in females and a positive correlation with age only in males. This study used large-sample data to investigate the role of sex as a confounding factor in the relationship between the oral microbiome and frailty for the first time, showing that, when stratified by sex, the features of the oral microbiome change with age and differ among frail elderly individuals [67]. However, in regard to sex-related subgroup analysis, the majority of previous studies have not revealed any appreciable variations in the oral microbiome of healthy older people. Future independent research is needed to confirm the connection between sex and the oral microbiome to better imply that the oral microbiome could be a useful predictor of increased risk for frailty.

Overall, alterations in older persons' oral microbiomes are linked to frailty, and some research points to a distinct independent relationship. However, the nature of cross-sectional studies limits the exploration of a causal relationship between the two. Since frailty is a description of a systemic condition, few studies have attempted to explain the link between frailty and systemic conditions with a single common mechanism. Instead, most studies focus on the microbiome in age-related diseases, as both aging and frailty increase the risk of disease, and it is easier to model and more focused on investigating certain disorders.

#### Differences in the oral microbiome in age-related diseases

Age-related diseases refer to a group of conditions whose incidence significantly increases with advancing age [68]. Although the prevalence of various diseases increases with age, our goal is to examine the common age-related pathological disorders that most older people encounter. Here, we explore the relationships among the oral microbiome, sarcopenia, bone loss, and neurodegenerative disorders.

Numerous review papers and research studies have confirmed the causal link between neurodegenerative disorders and the oral microbiome. The basic viewpoints of these studies are similar [69–72]: By disrupting oral homeostasis, oral pathogens (such as *Porphyromonas gingivalis*) can produce virulence factors and pathology products locally or directly invade local tissues to induce neurological diseases. They can also cross the blood‒brain barrier through blood and nerve routes. Alternatively, by aggravating cerebrovascular injury and increasing systemic inflammatory indicators, these pathogens might cause indirect damage to brain tissue. The chronic inflammation and immunosenescence associated with age may be closely correlated with the prevalence of neurological disorders in the elderly [2].

Sarcopenia is a syndrome characterized by progressive and generalized loss of skeletal muscle mass and strength, with the risk of adverse outcomes such as physical disability, poor quality of life, and death [73]. A decrease in muscle strength is common among elderly individuals. Recently, literature reviews have proposed a bidirectional relationship between oral health (e.g. oral condition, bite force, chewing, tongue pressure, and swallowing) and sarcopenia [74]. Further mechanistic studies [75] have suggested that the correlation between the oral microbiome and muscle strength may originate from the nitrate-reducing microbial community in the mouth [76]. Humans largely rely on commensal bacteria in the oral cavity and gut to reduce ingested nitrate to bioactive nitrite, which is further reduced to nitric oxide (NO) in the circulation and other tissues, thereby increasing systemic NO bioavailability [77]. An individual's ability to benefit from ingested nitrate may be affected by a dysfunctional oral microbiome, which in turn may impact muscle volume and strength. Recently, a saliva-based multiomics three-stage cohort study was conducted to identify biomarkers associated with sarcopenia progression to help identify high-risk populations [78].

Research on the relationship between the oral microbiome and bone loss has focused mostly on the loss of alveolar bone in periodontal disease, where there is strong evidence that an unbalanced oral microbiome in diseased periodontal tissues plays a major role in local bone loss [79–82]. For example, lipopolysaccharides (LPS) from gram-negative bacteria (such as *Porphyromonas gingivalis* and *Aggregatibacter actinomycetemcomitans*) can directly stimulate osteoclast differentiation and activate inflammatory responses via the toll-like receptor 4/nuclear factor-κB (TLR4/NF-κB) signaling pathway, leading to bone loss [80]. In research related to osteoporosis (OP), patients with periodontitis who cannot maintain good oral hygiene have a 6.02-fold greater risk of developing OP compared to those without periodontitis [83], and this association increases progressively with the severity of periodontal disease [84]. Moreover, one meta-analysis [85] concluded that individuals with OP are also more likely to suffer from gingivitis. Therefore, there may be a bidirectional relationship between oral microbiome dysbiosis and bone loss. This association may be related to the distant spread of oral pathogens, the interaction of the oral-gut axis, and the promotion of systemic inflammation by oral dysbiosis [86], but the specific mechanisms require further exploration. Future research into the directionality and mechanisms of this connection will require well-planned prospective cohort studies and animal experimentation.

Alterations in the oral microbiome observed in systemic frailty and age-related diseases may provide insights into their association with aging. As noted earlier, *Porphyromonas gingivalis* has been found to be enriched in Alzheimer’s disease, sarcopenia, and periodontitis, suggesting that chronic systemic low-grade inflammation may represent a shared cross-disease mechanism mediating these associations.

## Oral microbiome‒host interplay: inflammation, immunosenescence and unresolved directionality

After analyzing the foregoing data, we propose that aging, frailty, and age-related diseases may all independently alter the human oral microbiome. The oral microbiome in healthy individuals changes with age and is affected by 'unhealthy status' in unhealthy individuals. Conversely, oral microbiome dysbiosis may accelerate the aging process, leading to biological acceleration. Therefore, how can we explain the mechanisms underlying these associations?

### Established evidences: how microbes and inflamma-immuno aging fuel each other?

**Figure 1.**
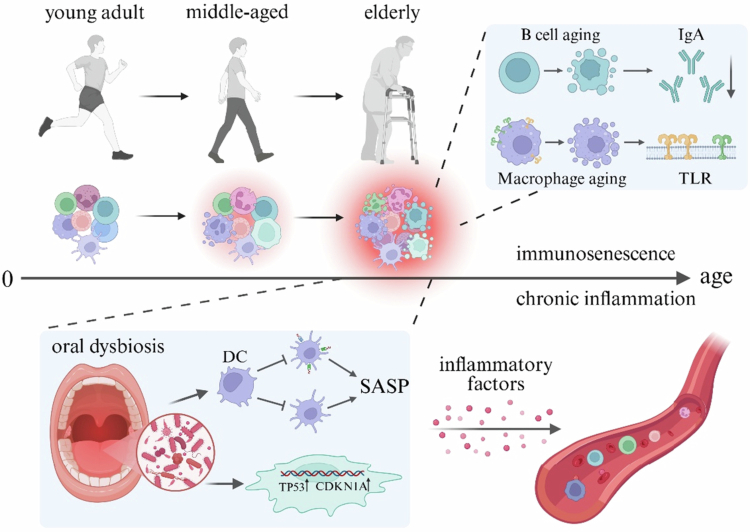
The relationship between inflammation-immune senescence and oral microbiome dysbiosis. With increasing age, B cell senescence is accompanied by a decrease in IgA secretion, and macrophage senescence is associated with changes in cell surface TLR expression. Immunosenescence leads to the proliferation of oral pathogens and opportunistic pathogens, resulting in oral dysbiosis. This further promotes changes in the expression of senescence-related genes in cells. The long-term effects of oral pathogens lead to DC senescence (decreased antigen-presenting function and maturation impairment), the development of the local SASP, and the development and maintenance of chronic inflammation. An arrow with a triangular head indicates a progressive relationship or changes within the same entity, while an arrow ending with a short vertical line signifies that the process is blocked or hindered. Abbreviations: IgA: immunoglobulin A; TLR: Toll-like receptor; DC: dendritic cell; SASP: senescence-associated secretory phenotype; TP53: tumor protein p53; CDKN1A: cyclin-dependent kinase inhibitor 1A. Created at https://BioRender.com.

Several studies have attempted to explain this correlation from the viewpoints of inflammation-related aging and immunosenescence. Among the hallmarks of aging, chronic inflammation plays a significant role [2]. Aging may influence the oral microbiome through continuous low-grade systemic inflammation, as there is enough evidence that the relationship between age and chronic inflammation persists even after adjusting for a number of confounding variables [87]. Immunosenescence refers mainly to the weakened ability of the immune system in older individuals to respond to external challenges, which usually complements inflammation-related aging. A chronic inflammatory state gradually develops with age, changing the local inflammatory environment of the oral cavity and resulting in changes in the oral microbiome. This may lead to an imbalance in oral homeostasis by fostering the growth of opportunistic infections and the proliferation of pathogenic bacteria. In turn, this imbalance promotes the maintenance and exacerbation of systemic inflammation. In addition, immunosenescence causes older people's gingival tissues to be less capable of eliminating infections, which could prolong the occurrence of chronic inflammation (Figure 1).

An example of how oral dysbiosis can lead to immunosenescence in animal experiments is that *Porphyromonas gingivalis* can induce premature senescence (autocrine), maturation defects, and impaired antigen-presenting function in mouse bone marrow-derived dendritic cells (DCs) through direct cellular invasion. It can also greatly amplify the senescence of bystander DCs [88] through paracrine effects by secreting inflammatory exosomes, which in turn promote the formation of the senescence-associated secretory phenotype (SASP). The SASP consists of an inflammatory secretome comprising proinflammatory cytokines, chemokines, and growth factors [89]. The chronic low-grade inflammation associated with aging is comparable to the increased levels of inflammatory factors associated with the SASP. The release of these exosomes can be eliminated by the antiaging agent rapamycin [88], suggesting that reversing the inflammatory state by regulating the microbiome in aging individuals may serve as a promising target for aging interventions (Figure 1).

In addition, studies exploring aging at the gene expression level have revealed that LPS derived from *Porphyromonas gingivalis* can significantly upregulate the expression of senescence-related genes (TP53 and CDKN1A) in human dental pulp stem cells in a time-dependent manner [90], which may exacerbate the susceptibility of older adults to periodontal disease.

At the same time, immune- and inflammation-related aging may mediate oral microbiome changes through certain key molecules and receptors. In a mice study [91], Mizuno et al. found that age-related changes in the oral microbiome (a significant decrease in the relative abundance of *Staphylococcus nepalensis*) may be driven by B cell senescence, which reduces immunoglobulin A secretion. Compared to young mice (2-month-old) [92], peritoneal macrophages from middle-aged mice (12-month-old) had impaired tolerance to repeated endotoxin stimulation from *Porphyromonas gingivalis*, and this difference in tolerance may partly stem from changes in the expression of TLR2 and TLR4, which can partly explain why older individuals are more prone to developing uncontrollable periodontal inflammation.

Beyond rodent-based studies, a broader research space may exist in nonhuman primate studies, such as rhesus macaques (*Macaca mulatta*). Current research has indicated that the oral microbiome composition of rhesus macaques is similar to that of humans [93], and studies of free-ranging animal populations (such as surveys of animals in independent habitats) may help reduce biases commonly seen in human studies, including lifestyle, diet, and medical interventions, providing a valuable model [94]. Janiak et al. analyzed a large dataset of oral, rectal, and genital swabs from 105 free-ranging rhesus macaques (aged 1 month to 26 years) and reported that age-related differences in the oral microbiome were primarily observed between infants (who were on a milk diet) and other age groups, whereas the microbial composition remained largely stable from adulthood to old age [94].

### Unresolved questions: who leads, who follows?

Current evidence links oral dysbiosis, chronic inflammation and immunosenescence in a self-amplifying loop, but the trigger remains elusive. *Porphyromonas gingivalis* may precipitate dendritic cell senescence and systemic SASP in rodents [88]; however, comparable human data are lacking. Determining whether deviation from a 'young' oral community precedes systemic inflammation in midlife or whether immune rejuvenation alone can reestablish eubiosis, help clarify who leads and who follows, and pinpoint where the cycle can be broken.

## The possibility of oral microbiome modulation to delay aging

Modifying the oral microbiome to prevent or postpone aging, enhance frailty, and lessen age-related illnesses is a significant next step for humans once the oral microbiome has been utilized to evaluate and detect aging.

First and foremost, maintaining good dietary and oral hygiene practices is essential for preserving a healthy oral microbiome and preventing age-related diseases. Proper dietary interventions may help maintain good oral and overall health [95]. A systematic review summarizing 19 studies on dietary patterns and oral health reported that dietary patterns such as the Mediterranean diet, plant-based diets, and diets rich in dairy products were generally associated with a lower risk of periodontitis than a Western diet high in proinflammatory foods (such as sugar, saturated fats, and refined carbohydrates) [96]. However, it should be noted that the majority of the included studies were cross-sectional, indicating associations rather than causalities between dietary patterns and periodontitis. This might be the case because healthier dietary patterns may encourage the growth of oral commensal bacteria (such as *Rothia*, *Actinomyces*, etc.) [97], whereas diets high in proinflammatory foods may promote microbial communities linked to oral inflammation [98]. Good oral hygiene and a higher number of teeth, as observed in long-lived populations, are beneficial for maintaining oral microbial diversity [99,100]. Maintaining oral hygiene can reduce the abundance of cariogenic and periodontal pathogens, decreasing the incidence of periodontitis and dental caries in the elderly [101] and thereby lowering the risk of age-related diseases such as Alzheimer's disease [72,102].

Second, the supplementation of probiotics and prebiotics is beneficial for restoring oral homeostasis. Oral probiotic supplementation is not only advantageous for the prevention and treatment of periodontitis [103] but also significantly improves systemic inflammation caused by oral dysbiosis. The reduction in chronic inflammation may in turn lower the risk of age-related diseases in various systems throughout the body [104–106]. Li et al. found that nitrate supplementation effectively alleviated cognitive deficits caused by long-term alcohol exposure in mice and reduced systemic neuroinflammation [107]. Compared to healthy mice, alcohol consumption led to increased abundances of *Escherichia*, *Lactobacillus*, *Pasteurella*, *Megasphaera*, and *Megamonas*, along with a decrease in taxa related to nitrate metabolism (e.g. *Streptococcus*). By preserving phyla such as *Firmicutes*, *Cyanobacteria*, *Proteobacteria* and *Actinobacteria*, nitrates could mitigate the notable differences in community structure between the alcohol group and the healthy group, decrease the increase in the Chao index caused by alcohol, and simultaneously increase the abundance of nitrate-metabolizing taxa (such as *Corynebacterium*) in the group that received alcohol plus nitrate supplementation [107]. Although the ability to benefit from nitrate supplementation varies among individuals [108], this study suggests that oral nitrate may improve cognitive deficits in aging by altering the oral microbiome [107].

Other potential interventions include oral microbiota transplantation (OMT), which corresponds to fecal microbiota transplantation (FMT). Studies related to FMT have shown that it can extend the lifespan of mice [109]. However, to date, OMT has been reported in only one case, where donor saliva from the patient's mother was used to effectively prevent the occurrence of oral mucositis after chemotherapy [110]. To date, no studies have explored the role of OMT in modulating aging.

In summary, few studies have focused on modulating the oral microbiome to delay aging. Most evidence has demonstrated that microbiome-targeted interventions may alleviate age-related disease burden rather than directly influencing biological aging itself. Future research should adopt longitudinal human cohorts and randomized controlled trials to clarify causal relationships between microbiome changes and aging trajectories. Mechanistic studies in animal or in vitro models are also needed to elucidate how microbial metabolites and immune signaling mediate systemic aging processes.

Moreover, the integration of multiomics approaches, such as metagenomics, metabolomics, and host transcriptomics, will be essential for uncovering the molecular pathways linking the oral microbiome and host aging. Given the substantial interindividual variation in microbial responses to diet and probiotics, precision interventions tailored to microbial or host profiles should be a key direction for future studies.

## Conclusions

In general, there appears to be a relationship between aging and the oral microbiome, whether in populations that are aging healthily or in those that are aging unhealthily, such as frailty. However, conclusions from different cross-sectional studies are inconsistent, with some studies reporting positive results in opposite directions and others reporting no significant results. Differences in how these studies define aging and health criteria, as well as the study populations' race, diet, lifestyle, inclusion and exclusion criteria, sampling techniques, and data analysis settings, are all responsible for this discrepancy. To minimize result bias caused by study design heterogeneity, it is necessary to strictly control for confounding factors and match the experimental and control groups. However, there are a significant number of unknown and uncontrollable confounding factors in human investigations. If necessary, animal studies, such as population studies of nonhuman primates [111], serve as a promising way to reduce biases caused by factors such as socioeconomic status, income levels, and dietary habits.

At the same time, most current microbiome studies are limited to species identification and descriptive research. In the future, it will be necessary to integrate multiomics studies, such as transcriptomics, proteomics and metabolomics, to further elucidate the functional differences and metabolic pathways produced by different microorganisms in their respective populations. This will facilitate the investigation of possible targets for therapies aimed against aging.

Currently, research on aging clocks is also in full swing. Aging clocks based on the gut microbiome have shown good predictive power for health and frailty status [112,113]. However, studies related to the oral microbiome are still in their infancy. As research on the oral microbiome progresses and multiomics technologies are integrated, an 'oral age' aging clock predicting human lifespan should be constructed based on oral microbiome phenotypes in the future.

The oral microbiome, a large microbial reservoir, is nevertheless a prospective target for aging therapies, despite the paucity of overall research. Since the microbiome is the largest symbiotic partner of humans, there is still opportunity for more research and advancement into the mysteries of aging and longevity, which have always piqued human interest.

## Data Availability

Data sharing is not applicable to this article as no new data were created or analyzed in this study.
